# Management of Chronic Myeloid Leukemia in Advanced Phase

**DOI:** 10.3389/fonc.2019.01132

**Published:** 2019-10-25

**Authors:** Massimiliano Bonifacio, Fabio Stagno, Luigi Scaffidi, Mauro Krampera, Francesco Di Raimondo

**Affiliations:** ^1^Department of Medicine, Section of Hematology, University of Verona, Verona, Italy; ^2^Division of Hematology With BMT, AOU Policlinico “Vittorio Emanuele”, University of Catania, Catania, Italy

**Keywords:** accelerated phase, blast phase, clonal evolution, molecular monitoring, allogeneic stem cell transplant

## Abstract

Management of chronic myeloid leukemia (CML) in advanced phases remains a challenge also in the era of tyrosine kinase inhibitors (TKIs) treatment. Cytogenetic clonal evolution and development of resistant mutations represent crucial events that limit the benefit of subsequent therapies in these patients. CML is diagnosed in accelerated (AP) or blast phase (BP) in <5% of patients, and the availability of effective treatments for chronic phase (CP) has dramatically reduced progressions on therapy. Due to smaller number of patients, few randomized studies are available in this setting and evidences are limited. Nevertheless, three main scenarios may be drawn: (a) patients diagnosed in AP are at higher risk of failure as compared to CP patients, but if they achieve optimal responses with frontline TKI treatment their outcome may be similarly favorable; (b) patients diagnosed in BP may be treated with TKI alone or with TKI together with conventional chemotherapy regimens, and subsequent transplant decisions should rely on kinetics of response and individual transplant risk; (c) patients in CP progressing under TKI treatment represent the most challenging population and they should be treated with alternative TKI according to the mutational profile, optional chemotherapy in BP patients, and transplant should be considered in suitable cases after return to second CP. Due to lack of validated and reliable markers to predict blast crisis and the still unsatisfactory results of treatments in this setting, prevention of progression by careful selection of frontline treatment in CP and early treatment intensification in non-optimal responders remains the main goal. Personalized evaluation of response kinetics could help in identifying patients at risk for progression.

## Introduction

Chronic myeloid leukemia (CML) is a myeloproliferative disorder characterized by the neoplastic transformation of the hematopoietic stem cell and the pathognomonic presence of the Philadelphia (Ph) chromosome arising from a reciprocal translocation between chromosomes 9 and 22. This balanced chromosomal alteration causes the fusion of the Abelson oncogene (ABL) from chromosome 9q34 with the breakpoint cluster region (BCR) on chromosome 22q11.2, *t*(9;22)(q34;q11.2) and induces the formation of a distinct chimeric BCR-ABL1 fusion gene, which in turn translates into a Bcr-Abl oncoprotein. This oncoprotein most frequently has a molecular weight of 210 kD (p. 210) and displays increased tyrosine kinase activity which causes growth factor independence and leukemic cell growth in hematopoietic cell lines, contributes also to the clonal evolution of the disease and leads to its evolution toward acute leukemia (1). CML usually presents in chronic phase (CP), characterized by the clonal expansion of mature myeloid cells. Indeed, all untreated patients will eventually progress to a lethal blast phase (BP) that is sometimes preceded by an accelerated phase (AP). The development of Tyrosine Kinase Inhibitors (TKIs) in the last 20 years has represented an outstanding revolution in the management and outcome of CML, and a paradigm for targeted therapy of cancer (2). Although life expectancy for patients diagnosed with CP-CML nowadays is similar to that of general healthy population (3), the onset of disease in advanced phase, or progression from CP to AP or BP following TKI failure still represent a complex challenge. In fact, advanced phases are typically resistant to treatment and have a worse prognosis, with death occurring from infection and bleeding complications similar to acute leukemia (4, 5).

In this review, we will focus on the biological characteristics of CML in advanced phase, the main results of the available treatments, the options for improving outcome, and, finally, we will briefly discuss on the optimal management of CP-CML in order to prevent disease evolution.

## Definition, Epidemiology and Biological Determinants of Advanced Phase

Definition of advanced phase is controversial (6). There are four main classifications, provided by the International Blood and Marrow Transplant Registry [IBMTR; (7)], the MD Anderson Cancer Center [MDACC; (8)], the World Health Organization [WHO; (9)], and the European LeukemiaNet [ELN; (10)]. Details about the different criteria are summarized in Table 1. The IBMTR criteria have been mostly used in studies of bone marrow transplantation (BMT) and are the most comprehensive, but include some parameters which are somewhat subjective (leukocytosis, thrombocytosis or splenomegaly unresponsive to treatment, or thrombocytopenia and anemia unrelated to therapy); the significance of these findings as determinants of advanced phase seems to shrink in the TKI era. One of the most striking differences among the classification systems is the threshold of blast percentage used for defining AP and BP (15–29% according to ELN and 10–19% according to WHO for AP; ≥30% according to ELN and ≥20% according to WHO for BP). These different cutoffs should be kept in mind when results of new strategies have to be evaluated. In a retrospective cohort of 809 patients treated with imatinib, those who had a blast count between 20 and 29% had better complete cytogenetic response (CCyR) and 3-year overall survival (OS) compared to patients with blasts ≥30%, thus validating the threshold proposed by ELN (11). Recently, the WHO has added “provisional” criteria for AP based on the response to TKI (absence of complete hematologic response to the first TKI, or absence of response to two sequential TKIs, or development of two or more BCR-ABL1 mutations while on TKI treatment), which require further validation (12).

**Table 1:** Definitions of accelerated and blast phase of chronic myeloid leukemia.

**Criteria**	**IBMTR**	**MDACC**	**ELN**	**WHO**
**ACCELERATED PHASE**				
Blasts (PB or BM)	10–29%	15–29%	15–29%	10–19%
Blasts plus promyelocytes (PB or BM)	>20%	≥30% with blasts <30%	≥30% with blasts <30%	–
Basophils (PB)	≥20%	≥20%	≥20%	≥20%
WBC	>100 × 10^9^/L	>100 × 10^9^/L	–	unresponsive to tx
Thrombocytopenia	<100 × 10^9^/L unrelated to therapy	<100 × 10^9^/L unrelated to therapy	<100 × 10^9^/L unrelated to therapy	<100 × 10^9^/L unrelated to therapy
Thrombocytosis	>1,000 × 10^9^/L unresponsive to tx	–	–	>1,000 × 10^9^/L unresponsive to tx
Anemia	Hb <8 g/dL, unresponsive to tx	–	–	–
Splenomegaly	Unresponsive to tx	Unresponsive to tx	–	Unresponsive to tx
Cytogenetics	CE, on treatment	CE, on treatment	ACA/Ph+ major route, on treatment	ACA/Ph+ major route, complex karyotype, or 3q26.2 abnormalities, at diagnosis; any new ACA/Ph+, on treatment
Response to TKI (provisional criteria)	–	–	–	Failure to achieve CHR to the first TKI, or Any hematological, cytogenetic, or molecular indication of resistance to 2 sequential TKIs, or Occurrence of ≥2 mutations in BCR-ABL1 during TKI therapy
**BLAST PHASE**				
Blasts (PB or BM)	≥30%	≥30%	≥30%	≥20%
Other	Extramedullary blast proliferation (apart from spleen)	Extramedullary blast proliferation (apart from spleen)	Extramedullary blast proliferation (apart from spleen)	Extramedullary blast proliferation, or large foci or clusters of blasts in the BM biopsy

*IBMTR, International Blood and Marrow Transplant Registry; MDACC, M.D. Anderson Cancer Center; ELN, European LeukemiaNet; WHO, World Health Organization; PB, peripheral blood; BM, bone marrow; CE, clonal evolution; ACA/Ph+, additional chromosome abnormalities in Philadelphia-positive cells; CHR, complete hematologic response*.

The incidence of advanced phase at diagnosis is not really defined. A proportion of patients with AP or BP features since first referral was reported as high as 6–11% and 8–16%, respectively, in large monocentric series (13, 14). National multicenter registries showed lower incidences, around or inferior to 5% each: specifically, advanced phase at diagnosis was reported in 3% of patients in France (2% AP and 1% BP) (15), 7% in Czech Republic and Slovakia (5% AP and 2% BP) (16), 5% in Turkey (4% AP and 1% BP) (17), 6% in Sweden (4% AP and 2% BP) (18), and 1% in Italy (19). Among the EUTOS population-based registry (2,904 patients), the incidence of AP and BP at diagnosis were 3.5% and 2.2%, respectively (20). These differences may be partly related to the different application of diagnostic procedures. For example, a bone marrow (BM) core biopsy has been found either essential or helpful in correctly defining the disease phase and in evaluating the presence of BM fibrosis, a feature related with advanced phase (21), however indication for performing routine BM biopsy at CML diagnosis is not provided by current guidelines (22, 23). A recent study, while assessing minimal residual disease in children with Philadelphia chromosome-positive acute lymphoblastic leukemia (Ph^+^-ALL), found that, in some patients, from 12 to 83% of nonmalignant B cells, T cells, and myeloid cells were positive for BCR-ABL1, suggesting that the translocation probably have occurred in a multipotent hematopoietic cell, thus concluding that these patients were actually affected by CML in blast crisis rather than *de-novo* Ph^+^-ALL (24). Although a similar study has not been performed in adult patients, the higher incidence of Ph^+^-ALL in the adult setting may suggests that presentation of CML in blast crisis could be more common than usually reported (25).

The incidence of progression from CP to blast crisis has dramatically decreased after the introduction of TKI therapy (26). In the pre-imatinib era progression rates were around 1.5–3.7% per year and decreased to 0.3–2.2% per year in the imatinib-based CML study IV (27). The same picture was seen in the imatinib arm of the pivotal IRIS trial, were the estimated 10-year cumulative incidence of blast crisis was 7.9% and appeared to be higher in the first 4 years after diagnosis, then decreasing around zero as soon as patients reached a molecular response (28). The introduction of 2nd generation TKI as frontline treatment of CP-CML further reduced the incidence of progression, although the difference vs. imatinib was statistically significant for the nilotinib arms only of the ENESTnd trial (0.7% for nilotinib 300 mg twice daily vs. 1.3% for nilotinib 400 mg twice daily vs. 4.8% for imatinib 400 mg daily at 5 years, *p* < 0.05 for both comparisons) (29) while there was a trend toward less progression rates in the dasatinib arm of the DASISION trial (3.0% for dasatinib 100 mg daily vs. 5.7% for imatinib 400 mg daily at 5 years) (30) and the bosutinib arm of the BFORE trial (1.6% for bosutinib 400 mg daily vs. 2.5% for imatinib 400 mg daily at 12 months) (31). In a non-academic healthcare setting investigated within the Swedish CML registry, the cumulative incidence of progression at 2 years from diagnosis was 4.3%. Of note, all patients undergoing progression had been treated with imatinib frontline, high-risk EUTOS score was associated to the risk of progression, and insufficient cytogenetic and/or molecular monitoring was found in 33% of them (32).

A detailed discussion about the mechanisms of evolution to advanced phase is beyond the scope of this article and there are many beautiful reviews on this topic (33–35). Here, we will focus on cytogenetic clonal evolution (CE) and on development of BCR-ABL1 mutations, two determinants of progression that may have a relevant impact on treatment choices and outcomes.

Cytogenetic CE is considered an AP-defining characteristic according to various classification systems (Table 1). A favorable outcome of patients displaying cytogenetic CE as the single feature of AP (i.e., not associated with high blast count, or other AP abnormalities) was demonstrated in patients treated with interferon (36), allogeneic BMT (37), imatinib (38) and 2nd generation TKI after imatinib failure (39). However, compared to patients with standard karyotype, those with cytogenetic CE have inferior responses to imatinib (40, 41) and the presence of additional chromosomal abnormalities (ACA) other than Ph chromosome at diagnosis are recognized as a warning feature by ELN (22). In the German CML Study IV the occurrence of trisomy 8 (+8), isochromosome i(17q), trisomy 19 (+19), or an extra copy of Ph (+Ph) had a striking unfavorable clinical impact on response to imatinib and prognosis, and these abnormalities were identified as “major route” ACA (42). In contrast, other cytogenetic aberrations, like the loss of Y-chromosome or other sporadic abnormalities, were called “minor route” ACA and they were considered as mere indicators of genomic instability instead of determinants of progression (43). In a comprehensive study from the MDACC, the unfavorable prognostic role of the isolated +8 was not confirmed and two previously considered minor-route ACA (3q26.2 rearrangement and monosomy 7/7q deletion) were associated to poor treatment response and dismal survival (44). Moreover, different ACA were associated to a lineage-specific progression to BP, being +8, 3q26.2 rearrangement, i(17q) and +19 significantly more common in myeloid BP, and−7/7q- more common in lymphoid BP (45). On this basis, a cytogenetic-based model for predicting the risk of progression to BP was recently proposed: patients without ACA represented the standard risk group, patients with +8, +Ph, or other single ACA the intermediate-1 risk group, patients with other complex ACA the intermediate-two risk group, and patients with isolated 3q26.2 rearrangement, −7/7q–, i(17q), or with these abnormalities in the context of a complex karyotype represented the high-risk group. This model predicted different probabilities of CE while on TKI treatment, with high-risk patients considered as candidates for transplant in first CP due to the high rate of rapid progression even when treated with 2nd generation TKI (46).

BCR-ABL1 kinase domain mutations have been detected in 26–37% of imatinib-naïve patients diagnosed in advanced phase (47), prompting the recommendation of performing mutation analysis in any case of AP or BP at diagnosis, but not in the large majority of CML patients presenting in CP (48). In patients failing imatinib, frequency and number of mutations correlate to the risk of progression to advanced phase, thus resistant mutations act at least in part as a determinant of disease evolution (49, 50). Moreover, time to progression to advanced phase and survival were significantly shorter in imatinib-resistant patients harboring BCR-ABL1 mutations compared to patients without detectable mutations (51). Mutational analysis is therefore recommended both in case of failure or suboptimal response to frontline treatment (48). Direct sequencing of the BCR-ABL1 gene is still the reference method for mutation detection, but its sensitivity is low. Using more sensitive techniques such as mass spectrometry (52) or next-generation sequencing (53), a higher number of low-level mutations were found in patients with inadequate response to their treatment, and predicted for lower rates of response to subsequent lines of treatment, especially if patients received TKIs to whom they were insensitive. Testing longitudinal samples of patients resistant to imatinib or 2nd generation TKIs, NGS technique revealed pathogenic BCR-ABL1 mutations in about half of cases 3 months before the same mutations could be detected by conventional sequencing (54), including cases harboring the highly resistant T315I mutation (55).

## The Role of TKI in Advanced Phase

Evidences about the optimal treatment of CML patients in advanced phase are much less solid than in CP patients, but the evolving concepts in the use of TKI have been also redirected in the management of AP and BP patients. Current indications of BCR-ABL1 inhibitors for CML in advanced phase are summarized in Table 2.

**Table 2:** Indications of currently available TKI in advanced phase of chronic myeloid leukemia.

**TKI**	**Relative potency against BCR-ABL1**	**Other targets**	**Indications in AP**	**Indications in BP**
Imatinib	1 (reference)	PDGFR > c-KIT	AP-CML or BP-CML	
Nilotinib	≈25	PDGFR	AP-CML with resistance of intolerance to prior therapy including imatinib	Not indicated
Dasatinib	≈325	Src > BTK > PDGFR > c-KIT	AP-CML or BP-CML with resistance of intolerance to prior therapy including imatinib	
Bosutinib	≈15	BTK > Src	AP-CML or BP-CML previously treated with one or more TKI (s) and for whom imatinib, nilotinib and dasatinib are not considered appropriate treatment options (EMA) AP-CML or BP-CML with resistance or intolerance to prior therapy (FDA)	
Ponatinib	≈900	PDGFR > VEGFR2 > Src > c-KIT	AP-CML or BP-CML who are resistant to dasatinib or nilotinib; who are intolerant to dasatinib or nilotinib and for whom subsequent treatment with imatinib is not clinically appropriate; or who have the T315I mutation (EMA) AP-CML or BP-CML resistant or intolerant to prior TKI therapy (FDA)	

*AP, accelerated phase; BP, blast phase; PDGFR, Platelet-derived Growth Factor Receptor; BTK, Bruton's Tyrosine Kinase; TKI, Tyrosine Kinase Inhibitors; EMA, European Medicine Agency; FDA, Food and Drug Administration*.

### Imatinib

Imatinib was the first targeted agent used in patients with advanced disease. Main results of imatinib clinical trials in AP and BP patients are reported in Tables 3, 4.

**Table 3:** Imatinib in accelerated phase of chronic myeloid leukemia.

**Study**	**Patients n^**°**^**	**AP definition**	**ACA/Ph+ patients**	**Hematologic response**	**Cytogenetic/molecular response**	**Survival**
Kantarjian et al. (56)	200	IBMTR criteria (modified)	41%	CHR 80%	MCyR 35% CCyR 24%	18-mo OS 73%
Talpaz et al. (57)	181	ELN criteria	excluded	HR 69%	MCyR 24%, CCyR 16%	1-year PFS 59% 1-year OS 74%
Kantarjian et al. (58)	176	ELN criteria	61% (36% with CE only)	CHR 82%	MCyR 49% CCyR 43%	4-years OS 53%
Palandri et al. (59)	111	ELN criteria	22%	CHR 71%	MCyR 30% CCyR 21%	7-years PFS 36% 7-years OS 43%
Jiang et al. (60)	87	WHO criteria	44% (11% with CE only)	CHR 85%	MCyR 49%, CCyR 47%, MMR 34%	6-years PFS 48% 6-years OS 51%
Rea et al. (61)	42	ELN criteria	62% (38% with CE only)	CHR 87%	MCyR 74% CCyR 60% MMR 45%	2-years PFS 87% 2-years OS 88%
Ohanian et al. (62)	30	ELN criteria	33%	CHR 97%	MCyR 83% CCyR 80% MMR 63%	3-years PFS 96% 3-years OS 87%
Furtado et al. (63)	139	MDACC criteria	29%	n.a.	MCyR 55%, CCyR 48% MMR 19%	5-years OS 66%

*AP, accelerated phase; IBMTR, International Blood and Marrow Transplant Registry; ELN, European LeukemiaNet; WHO, World Health Organization; MDACC, M.D. Anderson Cancer Center; ACA/Ph+, additional chromosome abnormalities in Philadelphia-positive cells; CE, clonal evolution; HR, hematologic response; CHR, complete hematologic response; MCyR, major cytogenetic response; CCyR, complete cytogenetic response; MMR, major molecular response; OS, overall survival; PFS, progression-free survival*.

**Table 4:** Imatinib in blast phase of chronic myeloid leukemia.

**Study**	**Patients** **n^**°**^**	**Daily dose of imatinib**	**Hematologic response**	**Cytogenetic response**	**Median survival**
Druker et al. (64)	58 (38 My-BP, 20 Ly-BP)	300–1000 mg	My-BP: HR 55%, CHR 11% Ly-BP: HR 70%, CHR 20%	MCyR 12% CCyR 9%	n.a.
Sawyers et al. (65)	229	400 or 600 mg	HR 67% CHR 15%	MCyR 16% CCyR 7%	6.9 months
Kantarjian et al. (66)	75	300–1000 mg	HR 52% CHR 21%	MCyR 11% CCyR 7%	6.5 months
Wadhwa et al. (67)	21	400 mg	HR 29%	n.a.	6.5 months
Sureda et al. (68)	30	600 mg	HR 60% CHR 33%	MCyR 3% CCyR 0%	10 months
Palandri et al. (69)	92 (72 My-BP, 20 Ly-BP)	600 mg	My-BP: HR 47%, CHR 24% Ly-BP: HR 60%, CHR 35%	MCyR 12% CCyR 10%	7 months

*My-BP: myeloid blastic phase; Ly-BP: lymphoid blastic phase (or de-novo Philadelphia-chromosome positive acute lymphoblastic leukemia); HR: hematologic response; CHR: complete hematologic response; MCyR: major cytogenetic response; CCyR: complete cytogenetic response*.

Early studies in AP-CML patients not previously exposed to other TKIs showed that imatinib determined 60–85% rates of complete hematologic response (CHR, 16–45% of complete cytogenetic response (CCyR) and 19–34% of major molecular response (MMR) (56–60, 63). OS ranged from 74% at 12 months to around 40–50% at 5–7 years, an outcome clearly less favorable as compared to CP patients.

Notably, these patients were mainly in late AP, as a result of progression from CP treated for years with chemotherapy or interferon. Studies in newly diagnosed AP-CML patients treated with imatinib frontline showed higher rates of CCyR (60–80%) and MMR (45–63%), and better survival (61, 62). Other reasons for the different results observed in these trials might reside in the criteria of patient selection (including or not patients with 10–15% blasts in blood or marrow) and the proportion of patients defined as AP-CML due to hematologic criteria, cytogenetic criteria, or both. Patients with CE at diagnosis but lacking hematologic signs of progression show a more favorable response to imatinib (40), while patients presenting with CE associated to hematologic criteria of AP have a worse outcome (61).

Efficacy rates of imatinib in BP-CML were around 50–70% for hematologic response (defined as return to CP, i.e., blasts <30%), 15–35% for CHR (defined as blasts <5%, normalization of blood counts and absence of extramedullary disease), and <10% for CCyR (64–69). However, these responses were largely transient, although the achievement of some degrees of cytogenetic response correlated to a better outcome. In a large international study and in a multicentric Italian study by the GIMEMA CML Working Party the median survival was 6 months for patients without major cytogenetic response (MCyR) and ranged from 12 to 20 months for patients achieving this response (65, 69). Overall, the benefit of imatinib single-agent was inferior in BP than in AP patients, since none of the BP-CML trials demonstrated a median OS longer than 1 year.

### Nilotinib

Nilotinib is a 2nd generation TKI with greater potency and BCR-ABL1 selectivity, and is effective against the majority of BCR-ABL1 mutants which confer resistance to imatinib. Main results of nilotinib clinical trials in AP and BP patients are reported in Tables 5, 6.

**Table 5:** 2^nd^/3^rd^ generation TKI in accelerated phase of chronic myeloid leukemia.

**Study**	**TKI**	**Patients** **n^**°**^ (R/I)**	**Hematologic response**	**Cytogenetic/molecular response**	**Survival**
**AFTER FAILURE OF IMATINIB AND/OR OTHER TKI**					
Kantarjian et al. (70)	Nilotinib 50–1,200 mg (phase I study)	56 (100%/0%)	HR 74% CHR 46%	MCyR 27% CCyR 14%	n.a.
Giles et al. (71)	Nilotinib 400 mg b.i.d.	21 (86%/14%)	HR 29%	MCyR 12% CCyR 0%	6-mo PFS 57% 1-year OS 80%
le Coutre et al. (72)	Nilotinib 400 mg b.i.d.	137 (80%/20%)	CHR 31%	MCyR 32% CCyR 21%	2-years PFS 33% 2-years OS 70%
Nicolini et al. (73)	Nilotinib 400 mg b.i.d.	181 (82%/18%)	CHR 22%	MCyR 19% CCyR 11%	18-mo OS 81%
Talpaz et al. (74)	Dasatinib 15–240 mg (phase I study)	11 (82%/18%)	HR 82% CHR 45%	MCyR 27% CCyR 18%	n.a.
Apperley et al. (75)	Dasatinib 70 mg b.i.d.	174 (93%/7%)	CHR 50%	MCyR 40% CCyR 33%	2-years PFS 46% 2-years OS 72%
Kantarjian et al. (76)	Dasatinib 70 mg b.i.d. (*n =* 159) or dasatinib 140 mg daily (*n =* 158)	317 (73%/27%)	CHR 47–52%	MCyR 39–43% CCyR 32–33%	2-years PFS 51–55% 2-years OS 63–72%
Gambacorti-Passerini et al. (77)	Bosutinib 500 mg daily	79 (86%/14%)	HR 57%	MCyR 40%	4-years OS 59%
Cortes et al. (78)	Ponatinib 45 mg daily	83 (92%/8%)	HR 55%	MCyR 39% CCyR 24%	1-year PFS 55% 1-year OS 84%
**AT DIAGNOSIS, TKI-NAÏVE**					
Ohanian et al. (62)	Nilotinib (*n =* 16) or dasatinib (*n =* 5)	21	CHR 95%	CCyR 90% MMR 76% MR^4.5^ 50%	3-years PFS 90% 3-years OS 95%
Jiang et al. (79)	Nilotinib/dasatinib	101	n.r.	EMR 62–65%	3-years PFS 78–95% 3-years OS 92–97%
Balsat et al. (80)	Nilotinib 300–400 mg b.i.d. (*n =* 39) or dasatinib 100–140 mg daily (*n =* 27)	66	CHR 97%	CCyR 84% MMR 70% DMR 50%	7-years PFS 83% 7-years OS 87%
Masarova et al. (81)	Nilotinib 400 mg b.i.d.	22	CHR 73%	CCyR 73% MMR 73% MR^4.5^ 55%	5-years PFS 91% 5-years OS 84%

*R/I, resistant/intolerant; HR, hematologic response; CHR, complete hematologic response; MCyR, major cytogenetic response; CCyR, complete cytogenetic response; MMR, major molecular response; EMR, early molecular response; DMR, deep molecular response (MR^4^ or better); OS, overall survival; PFS, progression-free survival*.

**Table 6:** 2^nd^/3^rd^ generation TKI in blastic phase of chronic myeloid leukemia, after imatinib failure.

**Study**	**TKI**	**My-BP**				**Ly-BP**			
		**Patients n^**°**^**	**HR**	**MCyR**	**Survival**	**Patients n^**°**^**	**HR**	**MCyR**	**Survival**
Kantarjian et al. (70)	Nilotinib	24	42%	21%	n.a.	9	33%	11%	n.a.
Giles et al. (82)	Nilotinib	105	60%	38%	32%@2-years	31	59%	52%	10%@2-years
Nicolini et al. (73)	Nilotinib	133	21%	14%	62%@1-year	50	28%	36%	66%@1-year
Talpaz et al. (74)	Dasatinib	23	61%	35%	n.a.	10	80%	80%	n.a.
Cortes et al. (83)	Dasatinib	109	34%	33%	38%@2-years	48	35%	52%	26%@2-years
Saglio et al. (84)	Dasatinib	149	28%	27%	24–28%@ 2-years	61	38%	46%	16–21%@ 2-years
Cortes et al. (78)	Ponatinib	52	29%	19%	n.a.	10	40%	40%	n.a.
		**R/I to dasatinib or nilotinib**				**T315I mutation**			
		**Patients n°**	**HR**	**MCyR**	**Survival**	**Patients n°**	**HR**	**MCyR**	**Survival**
Cortes et al. (78)	Ponatinib	38	32%	18%	29%@1-year	24	29%	29%	29%@1-year
		**Prior imatinib only**				**R/I to imatinib plus dasatinib and/or nilotinib**			
		**Patients n°**	**HR**	**MCyR**	**Survival**	**Patients n°**	**HR**	**MCyR**	**Survival**
Gambacorti-Passerini et al. (77)	Bosutinib	36	38%	50%	28%@4-years	28	15%	21%	17%@4-years

*My-BP, myeloid blastic phase; Ly-BP, lymphoid blastic phase (or de-novo Philadelphia-chromosome positive acute lymphoblastic leukemia); HR, hematologic response; MCyR, major cytogenetic response; R/I, resistant/intolerant*.

Two main trials investigated the role of nilotinib in AP-CML patients failing imatinib: a phase 2 registration study (72) and a phase 3b expanded-access study recruiting also patients resistant/intolerant to dasatinib or after stem cell transplant (73). In both studies the median duration of CML was around 5–6 years and median duration of previous imatinib treatment was more than 2 years. As expected for a heavily pretreated population, only a proportion of patients obtained clinical meaningful benefits: sustained CHR and CCyR were achieved by 22–31 and 11–21% of patients, respectively. Response rates were slightly better for patients who switched from imatinib due to intolerance than for resistance (72).

In two contemporary trials (a registration phase 2 study and an expanded access phase 3b study) nilotinib 400 mg twice daily was administered to BP-CML resistant (82%) or intolerant (18%) to imatinib (73, 82). CHR rates were 7–24 and 14–41% in myeloid and lymphoid BP-CML, respectively. Cytogenetic responses were obtained rapidly, but were transient. Few patients received an allogeneic stem cell transplant (SCT) after nilotinib. OS was similar in the two studies (10 months for myeloid BP-CML and 8 months for lymphoid BP-CML). Nilotinib was not further developed in BP-CML and did not receive regulatory approval for this setting of patients.

### Dasatinib

Dasatinib is a 2nd generation multitargeted inhibitor of BCR-ABL1 and SRC-family kinases, which differs from imatinib in its ability to bind to both the active and inactive conformations of the ABL kinase. Dasatinib is 325-fold more potent than imatinib against wild-type BCR-ABL1 and is effective against the majority of BCR-ABL1 mutants. Main results of dasatinib clinical trials in AP and BP patients are reported in Tables 5, 6.

Dasatinib was studied in a series of clinical trials in patients with CML in all phases of disease after resistance or intolerance to imatinib (the SCR/ABL Tyrosine Kinase Inhibition Activity Research Trial of Dasatinib [START] program). Results of the phase 2 study in AP-CML (START-A) were initially reported on the first 107 patients (85) and then on the full population of 174 patients (75). These patients had a long history of CML prior to dasatinib therapy (median 6.5 years) and were extensively pretreated with high doses of imatinib (59%) and multiple other therapies for CML, including transplant (18%). More than 50% of patients had BCR-ABL1 mutations. Hematologic and cytogenetic responses were obtained both in mutated and unmutated patients, except for the T315I mutation. CCyR was achieved by one third of the overall population. A randomized phase 3 trial (CA180-035) compared two schedules of dasatinib administration (70 mg twice daily vs. 140 mg once daily) in a large population of patients with CML in advanced phase after imatinib failure, including 317 AP-CML patients (76). Efficacy rates were similar across the two groups and consistent with the previous phase 2 study (75). Single daily 140 mg dose of dasatinib was associated with a better safety profile. A recent long-term update showed that the estimated 5-years OS rates were 45 and 57% for patients randomized to 140 mg once daily vs. 70 mg twice daily, respectively. Although numerically higher, the different OS rate does not suggest a higher efficacy of the twice daily regimen, but it is likely to be related to the subsequent therapies, since only 40 patients (13% of the original cohort) continued to receive their assigned treatment beyond 5 years (86).

Patients with myeloid or lymphoid BP-CML were enrolled in two parallel phase 2 studies (START-B/START-L) and received dasatinib at the dose of 70 mg twice daily with dose escalation to 100 mg twice daily in case of inadequate response (83, 87). A total of 157 patients were enrolled, all after imatinib failure (52% received imatinib at 800 mg daily dose). Of note, 42% of myeloid and 65% of lymphoid BP-CML had BCR-ABL1 mutations. McyR rate was inferior in myeloid than in lymphoid BP-CML (34 vs. 52%, respectively) but the median duration of response was higher (16.8 vs. 4.1 months) and translated in a longer median OS (11.8 vs. 5.3 months). As previously mentioned, a large randomized study compared two different schedules of dasatinib in advanced CML patients, including 209 BP-CML patients (84). Efficacy was similar in the two treatment arms and, consistently with the previous phase 2 study, patients with lymphoid BP-CML resulted to have higher cytogenetic responses than myeloid BP-CML (MCyR 46 vs. 27%, CCyR 37 vs. 18%, respectively) but these responses were transient. Median OS was 7.7–7.9 months for myeloid BP-CML and 9-11.4 months for lymphoid BP-CML. As in AP-CML patients, also in BP-CML population the once daily regimen was better tolerated, resulting in fewer dose adjustments and fewer treatment interruptions.

Overall, these studies demonstrated that dasatinib could determine hematologic and cytogenetic responses in a relevant proportion of patients with a long history of CML and extensive pre-treatments. No studies formally addressed the role of dasatinib as frontline treatment in BP-CML patients, although it is likely that these patients could benefit of more potent TKI treatment earlier rather than after failure of other therapies.

### Nilotinib and Dasatinib in AP-CML Patients, Frontline

In a monocentric series of 51 patients with AP features at the time of diagnosis, frontline treatment with nilotinib (*n* = 16) or dasatinib (*n* = 5) was superior to imatinib (*n* = 30) although the difference was not statistically significant. CCyR and MMR rates were 80 and 63% in the imatinib group, and 90 and 76% in the 2nd generation TKI group, respectively. The estimated 3-year OS was 87% with imatinib and 95% with 2nd generation TKIs (62). A retrospective comparison of 101 AP-CML and 656 CP-CML treated with frontline imatinib (*n* = 660), nilotinib (*n* = 85), or dasatinib (*n* = 11) showed that an early molecular response (i.e., BCR-ABL1 <10% at 3 months) was attained by 62–65% of AP-CML, similar to Sokal high-risk (58%) and Sokal intermediate-risk (66%) patients, but inferior to Sokal low-risk (83%) CP-CML patients. With a median follow-up of 39 months PFS and OS were 78 and 92% for AP-CML with blasts ≥15%, while the corresponding survival rates in AP-CML with basophils ≥20% were 95 and 97% (79). Preliminary results of two studies employing 2nd generation TKI in newly diagnosed AP-CML patients were recently presented. In a French multicenter cohort of 66 patients, treatment with nilotinib (*n* = 39) or dasatinib (*n* = 27) achieved CHR, CCyR, and MMR rates of 97, 84, and 70%, respectively. Long-term survival was excellent, with 7-year PFS and OS rates of 83.4 and 87.1%, respectively. Survival was similar in the group of 33 patients classified as AP for cytogenetic clonal evolution only and in the group of 33 patients with hematologic AP features, but the first group had a significantly higher probability of attaining a deep molecular response (DMR, MR^4^ or better, 66 vs. 33%, respectively) (80). Similar results were reported in 22 patients treated at the M.D. Anderson Cancer Center with nilotinib 400 mg twice daily. Rates of cytogenetic and molecular response were high, but 18% of patients lost their best achieved response while on study due to acquired BCR-ABL1 mutations. One electively discontinued nilotinib after a sustained DMR lasting for 107 months. After a median follow-up of 5.7 years, the estimated 5-year OS was 84% (81). Overall, these studies showed that nilotinib and dasatinib had similar efficacy in TKI-naïve AP-CML patients, probably superior to imatinib. Of note, in all these studies more than 50% of patients reached a DMR, demonstrating that an early treatment with potent TKI can counterbalance the negative prognostic impact of the advanced disease.

### Bosutinib

Bosutinib is a dual Src/Abl 2nd generation TKI. Its activity and tolerability was studied in a phase 1/2 trial enrolling CML patients in all phases of disease with resistance/intolerance to imatinib only or resistance/intolerance to imatinib plus dasatinib and/or nilotinib (77). Patients in AP-CML were 79. CHR and CCyR rates were 33 and 31%. Cytogenetic responses were higher in patients treated with bosutinib after imatinib only than in patients receiving bosutinib in ≥3rd line of treatment (MCyR 48 vs. 27%, CCyR 35 vs. 23%, respectively). Responses were durable in around 50% of patients. Considering patients receiving 2nd generation TKI in second-line only, 4 years OS rate with bosutinib (66%) compared favorably to the 60–70% 2-years OS rate of nilotinib (72) and dasatinib (76). Patients in BP-CML were 64, including 23 myeloid BP-CML, 10 lymphoid BP-CML and 31 BP-CML of unspecified lineage. CHR and CCyR rates were 28 and 22% and responses were higher in patients treated with bosutinib after imatinib only than in patients receiving bosutinib in ≥3rd line of treatment (MCyR 50 vs. 21%, CCyR 37 vs. 17%, respectively). Responses were achieved across various baseline BCR-ABL1 mutations both in AP and BP cohorts, except for patients with T315I (*n* = 13) for whom only one response was achieved. Median OS was 10.9 months with two patients (3%) still receiving bosutinib at 4 years.

### Ponatinib

Ponatinib is a potent 3rd generation BCR-ABL1 inhibitor, rationally designed to overcome resistance to other TKIs due to BCR-ABL1 mutations, including the T315I mutation. A large international phase 2 study (Ponatinib Ph-positive Acute lymphoblastic leukemia and CML Evaluation, PACE) was conducted to determine efficacy of ponatinib in heavily pretreated CML patients, resistant to many lines of treatment or harboring the T315I mutation (78). The study enrolled 449 patients, including 83 AP-CML and 62 BP-CML. In the AP-CML cohort 55% of patients had a major HR which was sustained after at least 1 year in half of them; MCyR was attained by 39% of patients and sustained after at least 1 year in 73% of them. Patients who received fewer previous treatments tended to have higher response rates. No single mutation conferring resistance to ponatinib was observed. Interestingly, estimated PFS and OS at 5 years were 22 and 49%, respectively (88). Seventeen AP-CML patients, stopped ponatinib due to resistance or intolerance: after ponatinib failure the outcome was poor, since one of four patients treated with SCT achieved MMR and none of the non-transplanted patients responded to subsequent therapy (89). In the BP-CML cohort 31% of patients had a major HR and 23% a MCyR by 6 months, but few patients maintained their best response after at least 1 year, and median OS was only 7 months, without substantial differences between patients resistant to dasatinib/nilotinib or with T315I mutation. Estimated OS at 3 years was 9% (88).

## TKI and Chemotherapy for the Treatment of Advanced Phase

Chemotherapy has been used for many years in BP-CML since it appeared logical to employ induction protocols designed for ALL or acute myeloid leukemia (AML) also in lymphoid and myeloid BP-CML, respectively (90). Rates of response of BP-CML to chemotherapy were around 30% but remission duration was short and intensive regimens determined high rates of myelosuppression and induction deaths (91–94). Less intensive treatments were associated to fewer toxicities but the outcome was similarly dismal (95). Median survival was 6–8 months and increased to 2 years or more in a subset of patients who responded to intensive chemotherapy and could subsequently proceed to allogeneic SCT (96). Hypomethylating agents (alone or in combination with low-dose chemotherapy) have been tried in patients with myeloid BP-CML and encouraging results have been observed especially in elderly patients (97, 98). In a retrospective comparison of different treatment modalities in 162 myeloid BP-CML patients, response rates were similar among patients treated with intensive chemotherapy or with decitabine, but the latter group had less toxicity and response duration was overall longer, with a highly significant benefit on survival when only older patients were considered (99).

After the advent of TKI, all these treatment modalities were tested in combination with BCR-ABL1 inhibitors. Main results of these studies are reported in Table 7. Overall, the majority of these trials was very small and did not produce convincing evidence that any chemotherapy combination was superior to TKI alone (100–103). Of note, in many cases patients continued to receive the same TKI they had been previously exposed while in the CP, and this could have limited the benefit of treatment combination. Outcome was better when patients were treated with chemotherapy and 2nd generation TKI (104, 105), or when only *de-novo* BP-CML were considered, a group of patients with a reported median OS of 3 years or longer (103, 106). Results of different treatment strategies were analyzed in a large monocentric series of 477 patients with primary BP-CML (15%) or progressed after CP-CML or AP-CML (85%). Initial therapy for blast crisis was represented by TKI alone, a combination of TKI and chemotherapy, and non-TKI treatment in 35, 46, and 19% of patients, respectively. TKI (alone or in combination) was mainly imatinib (189 patients), followed by dasatinib (110 patients), nilotinib, bosutinib, and ponatinib (<30 patients each). Rates of response were slightly superior in patients treated with TKI and chemotherapy, but benefits seemed to be better when comparing 2nd generation TKIs vs. imatinib than comparing the same TKI with or without chemotherapy. In the whole cohort, factors significantly associated to a better survival in multivariate analysis were lymphoid immunophenotype, *de-novo* BP-CML, age <58 years, and the realization of transplant after induction treatment (106). Smaller studies explored the combination of hypomethylating agents or other agents (omacetaxine) and TKI. Results of imatinib combination were disappointing (107, 108), partly because the majority of these patients had already failed imatinib (111). Combination of azacytidine and dasatinib, nilotinib or ponatinib determined better hematologic, cytogenetic and molecular responses, and OS of 2 years or more were observed (109, 110).

**Table 7:** TKI and chemotherapy or other agents in blastic phase of chronic myeloid leukemia.

**Study**	**Patients** **n^**°**^**	**Regimen**	**Hematologic response**	**Cytogenetic response**	**Median survival**
**COMBINATION WITH CHEMOTHERAPY**					
Rea et al. (100)	13 (ly-BP)	Imatinib plus VCR/DEX	CHR 85%	MCyR 46% CCyR 31%	n.r.
Fruehauf et al. (101)	16 (my-BP)	Imatinib plus MTZ/VP-16	HR 81% CHR n.r.	n.r.	6.4 months
Quintas-Cardama et al. (102)	19 (my-BP)	Imatinib plus IDA/ldARA-C	HR 74% CHR 47%	CCyR 16%	5 months
Deau et al. (103)	36 (my-BP)	Imatinib plus 7/3 ARA-C/DNM	HR 78% CHR 56%	MCyR 41% CCyR 30%	16 months
Milojkovic et al. (104)	4 (ly-BP / my-BP)	Dasatinib plus FLAG/IDA	CHR 100%	MCyR 100% CCyR 75% MMR 75%	n.r.
Strati et al. (105)	42 (ly-BP)	Imatinib or dasatinib plus HyperCVAD	CHR 90%	CCyR 58% MMR 50% CMR 25%	17 months
Jain et al. (106)	195 (ly-BP/my-BP)	Different TKI and chemo regimens	HR 64%	CCyR 29% MMR 16%	12 months
**COMBINATION WITH OTHER AGENTS**					
Oki et al. (107)	10 (my-BP)	Imatinib plus decitabine	HR 30% CHR 20%	MCyR 20%	3.5 months
Fang et al. (108)	12 (my-BP)	Imatinib plus omacetaxine	HR 91% CHR 58%	MCyR 91% CCyR 25%	75%@1-yr
Ghez et al. (109)	5 (my-PB)	Dasatinib or nilotinib plus azacytidine	CHR 100%	MCyR 80% CCyR 40% MMR 40%	24 months
Ruggiu et al. (110)	11 (my-BP)	Dasatinib or nilotinib or ponatinib plus azacytidine	CHR 71%	CCyR 43% MMR 15%	28.1 months

*Ly-BP, lymphoid blastic phase (in some trials also de-novo Philadelphia-chromosome positive acute lymphoblastic leukemia are included); My-BP, myeloid blastic phase; VCR/DEX, vincristine/dexamethasone; MTZ/VP-16, mitoxantrone/etoposide; IDA/ldARA-C, idarubicin/low-dose cytrarabine; ARA-C/DNM, cytarabine/daunorubicin; FLAG/IDA, fludarabine/cytarabine/G-CSF/idarubicin; HR, hematologic response; CHR, complete hematologic response; MCyR, major cytogenetic response; CCyR, complete cytogenetic response; MMR, major molecular response; CMR, complete molecular response*.

## Allogeneic SCT for the Treatment of Advanced Phase

The number of CML patients undergoing allogeneic SCT has dramatically reduced over years as a consequence of the TKI efficacy (112, 113). Current indications for allogeneic SCT in CML are listed in Table 8 (22, 23, 114). Pre-transplant treatment with TKI has no negative impact on transplant outcome (115). In AP-CML patients a randomized study showed that allogeneic SCT determined a superior outcome compared to imatinib in patients with at least one of the following characteristics: disease duration >12 months, anemia, and peripheral blood blasts >5% (60). In BP-CML a retrospective comparison showed a significantly higher 4-year OS for patients who underwent allogeneic SCT after TKI therapy as compared to those treated with TKI alone (47 vs. 10%; *p* < 0.001) (116). A consistent analysis from the CIBMTR reported a disease-free survival of 26–27% for AP-CML and 8–11% for BP-CML (117). OS rates ranged from 40 to 60% after 3–5 years from transplant (118, 119). Of note, advanced phase represents *in se* a risk factor for transplant outcome and was identified as one of the 5 predictors of survival after allogeneic SCT in CML by the EBMT, the other being donor type, patient age, donor/recipient sex, and time from diagnosis to transplant (120). A retrospective, indirect comparison among patients with T315I mutation who received ponatinib in the PACE study and those who were transplanted in the EBMT registry showed that in advanced phase allogeneic SCT represents an important and curative option, especially for BP-CML and might be considered early in patients developing clinical progression, after a trial of ponatinib therapy (121). In the setting of allogeneic SCT both myeloablative and non-myeloablative strategies have been used and which is the best regimen option still remains to be determined (122). After allogeneic SCT conventional molecular monitoring of BCR-ABL1 transcripts should be recommended whilst the role of a post-transplant TKI-therapy warrant further investigation.

**Table 8:** Indications for allogeneic stem cell transplant in chronic myeloid leukemia.

**Chronic phase**
Failure to respond to ≥3 TKIs Presence of T315I mutation and/or failure to ponatinib Presence of very high-risk cytogenetic features (e.g., isolated 3q26.2 rearrangement, −7/7q–, i(17q), or with these abnormalities in the context of a complex karyotype) Presence of recurrent severe cytopenias in response to treatment with different TKIs despite dose reduction and cytokine support
**Advanced phase**
AP patients with non optimal response to frontline TKI BP patients after acquisition of a second CP with TKI (plus optional chemotherapy) AP or BP patients progressing from a previous CP, after reacquistion of a second CP with TKI (plus optional chemotherapy)

## Emerging Treatment for CML in Advanced Phase

A growing genomic instability is the hallmark of advanced disease in CML and novel drugs, both targeting the BCR-ABL1-dependent and -independent mechanisms of resistance to TKI, are now considered in pre-clinical or clinical investigation and tested for combination efficacy (123–125).

Asciminib (ABL001) is a selective allosteric inhibitor of BCR-ABL1 (126). Differently from other TKIs, asciminib binds to the myristoyl pocket of ABL1 kinase, induces the formation of an inactive kinase conformation and blocks leukemic cells proliferation. It is currently tested in clinical trials for relapsed/refractory Philadelphia chromosome-positive leukemia patients (NCT02081378). The rational of asciminib design represents a major advance in target therapy since it allows a possible combination strategy (dual-drug targeting) with other BCR-ABL1 inhibitors (127).

Histone Deacetilase Inhibitors are small molecules that block HDAC enzymes involved in epigenetic modifications (128) and different HDAC isoforms have been found overexpressed in cancer cells. Since HDAC up-regulation has been associated with a reduction in both overall and disease-free survival, a possible role for HDAC inhibitors as antitumor drugs has been suggested (129). Pracinostat, vorinostat and panobinostat have been diversely evaluated in CML (130–134).

The BCL2-inhibitor venetoclax (ABT-199) has shown a BCL2-selective antagonism, presenting however a modest activity against CML progenitors when used as single agent but seeming to enhance imatinib cytotoxicity when used in combination (135, 136).

Following the evidence that JAK2 interacts with the ABL C-terminal, leading to its constitutive activation (137), JAK2 inhibitors have been combined with imatinib, nilotinib, and dasatinib with the aim of eliminating resistant CML cells and restoring TKI-sensitivity in resistant CML cell lines (138). On this basis, ruxolitinib is being evaluated in clinical trials alone or in combination with different TKIs in patients with advanced or resistant disease (NCT01702064, NCT02253277, NCT01751425, NCT01914484, NCT02973711).

Other molecules such as Aurora kinase inhibitors (tozasertib, danusertib, alisertib) have been used in advanced phases of CML and showed some degrees of clinical efficacy but also remarkable levels of toxicities (139–144).

## Open Questions and Perspectives

**Figure 1:**
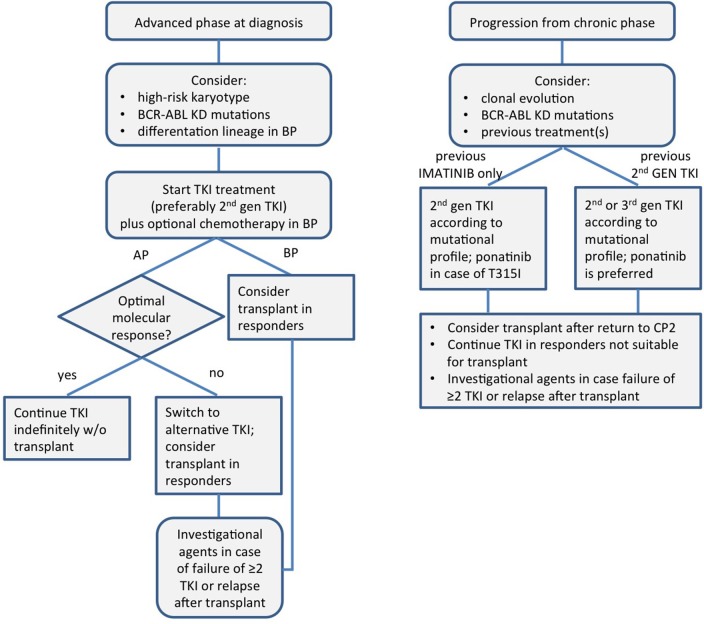
A schematic view of the modern management of chronic myeloid leukemia in advanced phase. KD, kinase domain; TKI, tyrosine kinase inhibitor; AP, accelerated phase; BP, blast phase; CP, chronic phase.

A schematic view of the modern management of CML in advanced phase is presented in Figure 1.

### How to Treat Patients in Advanced Phase in 2019

Earlier ELN treatment guidelines did not provide recommendations on treatment of advanced phase (10) or suggested allogeneic SCT for all patients in advanced phase preceded by a TKI treatment (145). More recent guidelines recognized the value of frontline TKI treatment without the need for subsequent transplant, especially for AP patients. In newly diagnosed AP patients treatment with TKI alone is recommended by ELN (22) and NCCN (23), and transplant is considered an option only for patients not achieving an optimal response. In the European Union, only imatinib 600 mg daily has a market authorization for frontline use in newly diagnosed patients in advanced phase, but its efficacy is limited by the development of BCR-ABL1 kinase domain mutations that occur more frequently in this setting than in CP. High efficacy of frontline treatment with nilotinib or dasatinib, including remarkable DMR rates, has been demonstrated in both retrospective (79) and prospective studies (80, 81). Interestingly, the magnitude of benefit of 2nd generation TKIs over imatinib in the randomized prospective trials on CP patients was more evident in high-risk patients, underlying thus the limits of imatinib in controlling a more aggressive disease (29–31).

For patients with *de-novo* BP, transplant is recommended in all patients after return to CP with TKI alone or in combination with chemotherapy (22, 114). No specific chemotherapy regimen can be suggested since studies are few, heterogeneous, and not controlled. In some retrospective series of patients treated with different modalities, the benefits of chemotherapy in addition to TKI were uncertain or minimal (32, 106). Moreover, in the setting of Ph^+^-ALL, the possibility of a rapid achievement of deeper rates of molecular response and long-term remission after TKI with minimal or no induction chemotherapy has been demonstrated (146–148). The use of frontline TKI followed by targeted immunotherapy (blinatumomab) is also actively investigated in prospective trials (NCT02744768, NCT03263572) and may represent an attractive option also for *de-novo* lymphoid BP-CML.

Disease progression to advanced phase while on TKI treatment has a worse prognosis than *de-novo* AP- or BP-CML. Treatment with alternative TKI, according to the BCR-ABL1 mutational status, participation in clinical trials, and evaluation for allogeneic SCT is recommended by current guidelines for all progressing patients (22, 23, 114).

In our opinion, the distinction between AP and high-risk CP features has limited value when 2nd generation TKIs are used in the frontline setting and early attainment of molecular response should be viewed as the only determinant for prognosis, irrespectively from disease characteristics at presentation. A chemo- and transplant-free approach for *de-novo* BP patients is also intriguing but there are very limited and indirect data to support such a strategy, and prospective trials are needed to address this issue. Patients progressing after multi-TKI failure represent a challenging setting in which further treatment with TKI alone has a limited value (149), and novel treatment options are eagerly awaited.

Of note, frontline treatment of advanced phase patients with TKI instead of chemotherapy and allogeneic SCT will further increase the economic burden of CML treatment, which still represents a relevant issue for sustainability of health systems (150).

### Which Are the Optimal Endpoints in the Treatment of AP/BP?

The optimal depth of response to frontline TKI at different time points is already well-defined for patients in CP (22, 23), but evidence for defining optimal response in advanced phases is still lacking. In earlier trials, it was observed that rates of MCyR often exceeded those of CHR in BP-CML patients (Tables 4, 5) and it was suggested that achievement of MCyR without CHR was associated to inferior survival (151). More recently, a direct correlation between the depth of response and survival was seen in a cohort of 386 patients with BP-CML: 5-year OS rates were 72, 34, 12, and 11% for patients with complete molecular response (CMR), MMR but not CMR, CCyR but not MMR, and HR only, respectively (152). Interestingly, no differences in survival were seen between patients who underwent or not to allogeneic SCT once they had obtained a CMR, reinforcing the concept that rapid kinetics of response to TKI therapy could allow long-term disease control even without transplant.

### Should Patients With Myeloid and Lymphoid BP-CML Receive Different Treatments?

Response to TKI is similar in myeloid and lymphoid BP-CML, although in the large retrospective experience of MDACC a lymphoid phenotype was associated with a better survival (106). Targeted immunotherapy, i.e., with the anti-CD33 antibody gemtuzumab ozogamicin in myeloid BP or the anti-CD19 bispecific antibody blinatumomab for lymphoid BP, may represent an attracting distinctive treatment strategy but reports are still anecdotal (153, 154). Another concern is about the utility of distinguishing lymphoid BP-CML from Ph^+^-ALL, since the differential diagnosis is often challenging. Patients with no preceding history of CP-CML may be considered as *de-novo* lymphoid BP-CML instead of Ph^+^-ALL if they have morphologic features of CML, such as left-shifted myeloid predominance, eosinophilia, and/or basophilia. The surface marker CD26 (dipeptidylpeptidase-IV) has been proposed as a specific marker of CML Leukemic Stem Cell (LSC) (155) and the assessment of CD26^+^ LSC in peripheral blood by flow cytometry has been suggested as a rapid tool for CML diagnosis (156) or as a marker for minimal residual disease during TKI treatment and treatment-free remission (157). Notably, in Ph^+^-ALL CD26 expression was found on LSC in patients with major BCR-ABL1 transcript encoding p210 BCR-ABL1 protein but not in patients with minor BCR-ABL1 transcript encoding p190 BCR-ABL1 protein (158). This finding may have therapeutic implications, since CD19-negative myeloid lineage relapses after blinatumomab have been observed in p210 Ph^+^-ALL patients as a result of the selection of preexisting CD19-negative malignant progenitor of myeloid origin (159). Although suggestive, these biological distinctions are not yet useful in guiding treatment selection and their clinical significance still remains to be elucidated.

### Is It Possible to Prevent Blast Crisis by the Optimization of Treatment in Chronic Phase?

Despite the groundbreaking results obtained with TKI in CP-CML, ~5–10% of CML patients eventually progress to advanced phase while on treatment. The mechanisms underlining TKI failure, disease progression and cytogenetic evolution remain largely unknown. Mutations in the ABL1 kinase domain, amplification of the BCR-ABL1 oncogene and high expression levels of the BCR-ABL1 mRNA represent BCR-ABL1 dependent mechanisms responsible for TKI failure (160). Then, uncontrolled BCR-ABL1 signaling leads to genetic instability and a more disorganized state until the anaplastic threshold is reached and other oncogenes ultimately lead to progression in a BCR-ABL1-independent way (161). Both quantitative and qualitative mechanisms can underlie the more aggressive behavior of CML clones expressing high BCR-ABL1 levels. According to the quantitative hypothesis, higher BCR-ABL1 transcripts translate into higher expression of the BCR-ABL1 oncoprotein and increased tyrosine kinase activity that would ultimately strengthen canonical BCR-ABL1-dependent signaling, resulting in a less responsive leukemic population. Higher BCR-ABL1 transcripts at diagnosis measured using *GUS* as a reference gene identified patients with inferior probability of response to frontline standard dose imatinib (162). On the other hand, the qualitative hypothesis assumes that higher BCR-ABL1 activity leads to “leakage” of BCR-ABL1 signaling to downstream targets that are usually not involved in BCR-ABL1-dependent transformation.

Many baseline factors at CML diagnosis have been correlated to a different probability of progression. Patients identified as high-risk by current prognostic models have a higher likelihood of disease transformation to AP-CML or BP-CML. In particular, the Eutos Long-Term Survival (ELTS) score identifies three risk groups with significantly different probabilities of death due to progression in advanced phase (163). Using the standard Sokal model, it was seen that within the same risk group younger patients had a higher risk of sudden progression and death (164). Biological variables associated to a high risk of progression included CIP2A levels (165), the expression levels of the polycomb group BMI1 gene (166), the activation of beta-catenin (167), specific gene signatures (168), and mutations in cancer-associated genes such as ASXL1, IKZF1, RUNX1, SETD1B, GATA2, MLL, and UBE2A (169). None of these variables has been extensively validated or is easily available in clinical practice.

The most relevant predictor of progression is the kinetics of response to treatment (170). It has been extensively demonstrated that not achieving a reduction <10% BCR-ABL1 after 3 months is linked to a higher risk of progression to advanced phase and reduced survival both with frontline imatinib and 2nd generation TKIs (29, 171–173). Measurement of the BCR-ABL1 transcript halving time during the first months of treatment may increase sensitivity and specificity of response measurement (174, 175). Finally, those patients who are regularly monitored according to guidelines have a lower risk of progression than patients monitored less frequently (176). However, sudden onset of BP despite adequate monitoring and apparently adequate response to TKI may occasionally occur (177, 178).

In our opinion, ELTS scoring system should be preferably used for frontline treatment decisions in all newly diagnosed CP-CML patients, and non low-risk patients should be considered for 2nd generation TKIs, or carefully monitored for early molecular response when imatinib is chosen as frontline treatment.

## Conclusion

Management of CML in advanced phase remains challenging. However, prognosis for patients diagnosed in AP improved clearly over years and presently the majority of patients with AP features at diagnosis can be managed as high-risk CP patients. Patients in blast crisis have inferior outcomes due to emergent resistance to TKI. Rational combination of TKI and chemotherapy or, preferably, novel agents including immunotherapy could improve remission rates and duration.

Frontline imatinib results challenged the concept of transplantation in CP-CML patients; nowadays the use of more potent TKI might modify the same concept also in patients presenting with advanced disease. However, optimal management of patients in CP represents the best way to avoid disease evolution and to allow a quite normal life duration for all patients.

Due to the limited evidences and the still numerous unmet needs, it would be desirable that a dedicated expert panel would provide updated recommendations for the management of CML in advanced phase.

## Author Contributions

All authors reviewed the literature, wrote the paper, and agree to be accountable for the content of the work.

### Conflict of Interest

MB declares research funding from Novartis and consultancy fees (advisory board honoraria) from Amgen, Incyte, Novartis, and Pfizer, outside the present work; FS and FD declare consultancy fees (advisory board honoraria) from Bristol Myers Squibb, Incyte, Novartis, and Pfizer, outside the present work. The remaining authors declare that the research was conducted in the absence of any commercial or financial relationships that could be construed as a potential conflict of interest.
